# The association between hydroxychloroquine treatment and cardiovascular morbidity among rheumatoid arthritis patients

**DOI:** 10.18632/oncotarget.23570

**Published:** 2017-12-15

**Authors:** Michael Shapiro, Yair Levy

**Affiliations:** ^1^ Department of Medicine E, Meir Medical Center, Kfar Saba, Israel; ^2^ Sackler Faculty of Medicine, Tel Aviv University, Tel Aviv, Israel

**Keywords:** hydroxychloroquine, cardiovascular morbidity, rheumatoid arthritis

## Abstract

**Objectives:**

To examine the independent effect of hydroxychloroquine (HCQ) treatment on cardiovascular morbidity among RA patients.

**Materials and Methods:**

A retrospective cohort study of RA patients treated at Meir medical center between 2003-2013 was conducted. Patients were divided into two groups, those who had been treated with HCQ during the course of their disease and those who had never received it. The two groups were compared for possible confounding factors. Study endpoints included arterial and venous thrombotic events.

**Results:**

A total of 514 suitable RA patients were identified, 241 HCQ-treated and 273 non-treated patients. Of the HCQ-treated patients, 32 (13.3%) suffered from cardiovascular events compared to 104 (38.1%) of the non-treated group. HCQ treatment had a significant protective effect for all cardiovascular events examined (HR = 0.456 CI 0.287 to 0.726) as well as arterial events alone (HR = 0.461 CI 0.274 to 0.778). A dose of 400 mg HCQ per day demonstrated a protective effect for any cardiovascular event (HR = 0.432 CI 0.243 to 0.768), while the lower dose of 200 mg per day showed no significant protective effect.

**Conclusions:**

The use of HCQ is independently associated with decreased risk for cardiovascular morbidity among RA patients, particularly with a higher dose of 400 mg per day. This newly demonstrated effect of HCQ should be considered in the overall management of RA.

## INTRODUCTION

Rheumatoid arthritis is a chronic inflammatory disease affecting approximately 1.3 million adults in the US alone [1]. The hallmark of the disease is an inflammatory process affecting peripheral joints, leading to deformity. In addition, about 40% of patients will incur various systemic manifestations leading to severe morbidity and mortality [2]. The risk factors for systemic disease include high levels of RF and anti-CCP markers and smoking [3].

The increased risk for cardiovascular disease (CVD) is one of the most severe systemic manifestations of RA. Several large studies showed that RA patients have 1.5 times the risk for myocardial infarctions (MI) and twice the risk for stroke than the general population [4, 5]. A large study of about 12 thousand RA patients compared to 58 thousand matched controls showed a 30% higher proportion of ischemic heart disease among RA patients [6].

For these patients, traditional cardiovascular disease (CVD) risk factors, such as diabetes, dyslipidemia and hypertension (HTN) play a significant role [4]. However, the increased CVD risk is attributed to systemic inflammation that is thought to promote accelerated atherosclerosis [5, 7, 8]. Moreover, there is strong evidence showing that inflammatory markers, such as CRP are independently associated with CV mortality and morbidity in RA [9].

In 2010, The European League Against Rheumatism) EULAR) published recommendations for cardiovascular risk management in patients with RA. The recommendations included recognition of RA as a condition with higher risk for CVD, especially for patients with a disease duration of more than 10 years, who have positive RF or anti-CCP titers and present with extra-articular manifestations. Additional recommendations included adequate control of disease activity, treatment with statins and ACE inhibitors and smoking cessation [10].

The current recommended therapy for RA is early, aggressive treatment at diagnosis in order to reduce the inflammatory process and prevent joint deformity. First line treatment usually combines limited steroid therapy and traditional disease-modifying anti-rheumatic drugs (DMARDS). The most commonly used drugs include methotrexate (MTX), hydroxychloroquine (HCQ) and sulfasalazine. The second line therapy includes a growing variety of new biological agents [11, 12].

Hydroxychloroquine, an anti-malarial drug, is one of the oldest drugs used in rheumatology. It is widely used as a first line therapy to treat RA and systemic lupus erythematosus (SLE), usually concomitant with other therapies. Lately, this drug has been losing favor and is frequently omitted, especially when biological agents are added [11].

Several studies examined the effects of HCQ on cardiovascular risk factors. A retrospective study, including over 600 RA patients treated with various combinations of DMARDs compared to prednisone alone, found a protective effect against cardiovascular morbidity with the combined therapy of HCQ, MTX and sulfasalazine. Due to its design and size, the study was unable to show a significant effect of HCQ treatment alone [13]. Three studies examining the effect of HCQ on lipid profile among patients with RA and SLE found a significant decrease in LDL and triglyceride levels, as well as an improvement in the LDL/HDL ratio [14–16]. HCQ was also found to have a protective effect from developing diabetes among RA patients [17]. Several studies on HCQ among SLE patients found a protective effect against atherosclerosis and venous thrombosis events [18]. In light of these results, this study was conducted to evaluate the effect of HCQ on cardiovascular morbidity among patients with RA.

## RESULTS

The patients’ characteristics for the treated vs the non-treated group are presented in Table 1.

**Table 1:** HCQ treated vs non treated patients’ characteristics

Characteristic	HCQ treated patients	Non-treated patients	Significance
**Demographics**			
Age ± SD (years)	68.4 ± 12.8	75 ± 12.1	*P <* 0.001
Gender (% female)	187 (77.6%)	209 (76.6%)	*P =* 0.916
**Disease severity markers**			
CRP (IQR) (mg/dl)	2.21 (0.6-2.7)	3.23 (0.69-3.3)	*P =* 0.030
RF (IQR) (IU/ml)	180.55 (5-169)	177.45 (5-158)	*P =* 0.932
Anti-CCP (IQR) (IU/ml)	165.06 (4-250)	118.03 (3.3-250)	*P =* 0.383
Disease duration (years)	11.3 ± 7.1	13.5 ± 9.1	*P =* 0.002
**Cardio-vascular risk factors**			
HTN	124 (51.5%)	164 (60.1%)	*P =* 0.051
Diabetes	98 (40.8%)	117 (42.9%)	*P =* 0.363
Hemoglobin A1C level ± SD (%)	7.1 ± 1	7.4 ± 1.1	*P =* 0.114
Smoking	66 (27.4%)	59 (21.6%)	*P =* 0.149
Obesity	73 (30.3%)	56 (20.5%)	*P =* 0.014
Previous cardiovascular disease	31 (12.9%)	22 (8.1%)	*P =* 0.082
Statin use	115 (47.7%)	120 (44.0%)	*P =* 0.425
Anti-aggregation therapy	90 (37.3%)	113 (41.4%)	*P =* 0.367
**Lipid profile**			
LDL ± SD (mg/dl)	100.2 ± 28.8	107.8 ± 29	*P =* 0.004
HDL ± SD (mg/dl)	57.3 ± 16.7	55.0 ± 15.8	*P =* 0.476
Triglyceride ± SD (mg/dl)	132.4 ± 62.1	134.6 ± 53.8	*P =* 0.806
**Other treatment**			
Azathioprine	1 (0.4%)	5 (1.8%)	*P =* 0.221
Sulfasalazine	75 (31.1%)	35 (12.8%)	*P <* 0.001
Prednisone	192 (79.2%)	198 (72.5%)	*P =* 0.078
MTX	154 (63.9%)	145 (53.1%)	*P =* 0.016
NSAIDs (constant therapy)	15 (6.2%)	20 (7.3%)	*P =* 0.726
Leflunomide	17 (7.1%)	10 (3.7%)	*P =* 0.112
Biological treatment	59 (24.5%)	32 (11.7%)	*P <* 0.001

SD = standard deviation, IQR = interquartile range, CRP = C-reactive protein, RF = rheumatic factor, HTN = hypertension, LDL = low density lipoprotein, HDL = high density lipoprotein, MTX = Methotrexate, NSAIDs=non-steroidal anti-inflammatory drug.

A total of 514 RA patients treated at Meir Medical Center from 2003 through 2013 were identified. Among these, 241 patients (46.9%) were treated with HCQ and 273 (53.1%) had never been treated with this medication.

As shown, both groups were predominately female (about 77%) and the HCQ-treated group were younger than the non-treated group (*P* < 0.001). Furthermore, the HCQ-treated group had significantly shorter RA disease duration (*P* = 0.002) and lower average CR*P* value (*P* = 0.03) compared to the treated group. The average anti-CCP level was not different between the groups. However, information regarding the anti- CCP levels was available only for 55 patients (10.7%), as the test was approved in Israel for all RA patients only in 2012.

Most of the cardiovascular risk factors were similar between the two groups. However, the HCQ-treated group were more obese (*P* = 0.014) and had significantly lower LDL levels (*P* = 0.004) while having higher rates of obesity.

In addition, patients treated with HCQ also received MTX (*P* = 0.016), sulfalazine (*P* < 0.001) and biological agents (*P* < 0.001) more often than the non-treated group. Only 6 patients were treated with azathioprine, most in the non-treated group.

The patients’ characteristics for patients treated with 400 mg per day and 200 mg a day of HCQ vs the non-treated group are presented in Table 2.

**Table 2:** Non-treated patients vs patients treated with 400 mg/day and 200 mg/day dose of HCQ characteristics

Characteristic	Non-treated patients	HCQ Treated 400 mg/day	Significance (400 mg vs non-treated)	HCQ Treated 200 mg/day	Significance (200 mg vs non-treated)
**Demographics**					
Age ± SD (years)	75 ± 12.1	66.6 ± 12.7	*P <* 0.001	71.6 ± 12.2	*P =* 0.028
Gender (% female)	209 (76.6%)	120 (76.4%)	*P =* 0.99	67 (79.8%)	*P =* 0.345
**Disease severity markers**					
CRP (mg/dl)	3.23 SD = 7	2.15 SD = 3.2	*P =* 0.025	2.3 SD=2.98	*P =* 0.244
RF (IU/ml)	177.45 SD = 396	200.7 SD = 429	*P =* 0.59	142.9 SD=302	*P =* 0.473
Anti-CCP (IU/ml)	118.03 SD = 192	178.9 SD = 209	*P =* 0.341	146 SD=180.6	*P =* 0.663
Disease duration (years)	13.5 ± 9.1	10.8 ± 6.5	*P <* 0.001	12.3 ± 8.2	*P =* 0.272

HTN	164 (60.1%)	76 (48.4%)	*P =* 0.021	48 (57.1%)	*P =* 0.703
Diabetes	117 (42.9%)	69 (43.9%)	*P =* 0.084	29 (34.9%)	*P =* 0.206
Hemoglobin A1C level ± SD (%)	7.4 ± 1.1	7.1 ± 0.96	*P =* 0.043	7.2 ± 1.1	*P =* 0.319
Smoking	59 (21.6%)	43 (27.4%)	*P =* 0.196	23 (27.4%)	*P =* 0.30
Obesity	56 (20.5%)	47 (29.9%)	*P =* 0.034	26 (31%)	*P =* 0.054
Previous cardiovascular disease	22 (8.1%)	21 (13.4%)	*P =* 0.095	10 (11.9%)	*P =* 0.280
Statin use	120 (44.0%)	74 (47.1%)	*P =* 0.547	41 (48.8%)	*P =* 0.454
Anti-aggregation therapy	113 (41.4%)	61 (38.9%)	*P =* 0.612	29 (34.5%)	*P =* 0.308

LDL ± SD (mg/dl)	107.8 ± 29	98.8 ± 27.1	*P =* 0.002	102.7 ± 31.6	*P =* 0.173
HDL ± SD (mg/dl)	55.0 ± 15.8	56.2 ± 16.6	*P =* 0.475	59.1 ± 16.8	*P =* 0.047
Triglyceride ± SD (mg/dl)	134.6 ± 53.8	133.1 ± 65	*P =* 0.806	131 ± 56.6	*P =* 0.601
**Other treatment**					
Azathioprine	5 (1.8%)	0 (0%)	*P =* 0.991	1 (1.2%)	*P =* 0.999
Sulfasalazine	35 (12.8%)	52 (33.1%)	*P <* 0.001	23 (27.4%)	*P =* 0.004
Prednisone	198 (72.5%)	127 (80.9%)	*P =* 0.062	65 (77.4%)	*P =* 0.478
MTX	145 (53.1%)	99 (63.1%)	*P =* 0.055	55 (65.5%)	*P =* 0.059
NSAIDs (constant therapy)	20 (7.3%)	9 (5.7%)	*P =* 0.690	6 (7.1%)	*P =* 0.999
Leflunomide	10 (3.7%)	16 (10.2%)	*P =* 0.010	1 (1.2%)	*P =* 0.469
Biological treatment	32 (11.7%)	35 (22.3%)	*P <* 0.001	18 (21.4%)	*P =* 0.007

Patients treated with a dose of 200 mg/day of HCQ were younger, had higher levels of HDL and were treated more with sulfasalazine and biological agents than non-treated patients.

Patients treated with 400 mg/day of HCQ were younger, had lower CRP and LDL levels and had shorter disease duration. They suffered more from HTN and obesity and were treated more with sulfasalazine, leflunomide and biological agents than non-treated patients.

Of the HCQ-treated patients, 32 (13.3%) suffered from any cardiovascular events compared to 104 (38.1%) of the non-treated group. A time dependent Cox- regression of these outcomes is presented in Table 3. Treatment with HCQ had a significant protective effect for any cardiovascular event with HR=0.456 (CI 0.287 to 0.726).

**Table 3:** Cox regression with time-dependent covariate of HCQ treated patients vs non-treated

	Significance	HR	95.0% CI for HR	
			Lower	Upper
Treatment	0.001	0.456	0.287	0.726
Age	0.037	1.019	1.001	1.036
Disease length	0.043	0.971	0.944	0.999
CRP (mg/dl)	0.000	1.108	1.053	1.167
LDL	0.000	0.988	0.981	0.995
Obesity	0.509	0.859	0.548	1.348
Sulfasalazine treatment	0.711	0.902	0.524	1.553
MTX treatment	0.103	0.722	0.489	1.068
Use of biological agents	0.364	0.726	0.364	1.449

Outcome of any Cardiovascular disease. HR –Hazard Ratio.

The effects of HCQ treatment on arterial events only are shown in Table 4. Treatment with HCQ had a significant protective effect for arterial events with HR = 0.461 (CI 0.274 to 0.778).

**Table 4:** Cox regression with time-dependent covariate of HCQ treated patients vs non-treated

	Significance	HR	95.0% CI for HR	
			Lower	Upper
Treatment	0.004	0.461	0.274	0.778
Age	0.176	1.013	0.994	1.033
Disease length	0.034	0.964	0.932	0.997
CRP (mg/dl)	> 0.001	1.122	1.060	1.188
LDL	0.001	0.986	0.978	0.994
Obesity	0.697	0.905	0.546	1.499
Sulfasalazine treatment	0.328	0.719	0.372	1.391
MTX treatment	0.196	0.746	0.478	1.164
Use of biological agents	0.326	0.664	0.293	1.503

Outcome: Arterial events only (MI, CVA and TIA).

The effects of HCQ treatment on venous events only are shown in Table 5. Treatment with HCQ had a significant protective effect for venous thrombotic events with HR = 0.386 (CI 0.155 to 0.961).

**Table 5:** Cox regression with time-dependent covariate of HCQ treated patients vs non-treated

	Significance	HR	95.0% CI for HR	
			Lower	Upper
Treatment	0.041	0.386	0.155	0.961
Age	0.040	1.036	1.002	1.071
Disease length	0.842	1.004	0.964	1.046
CRP (mg/dl)	0.043	1.094	1.003	1.194
LDL	0.106	0.990	0.978	1.002
Obesity	0.523	0.758	0.325	1.772
Sulfasalazine treatment	0.259	1.629	0.698	3.799
MTX treatment	0.318	0.701	0.349	1.408
Use of biological agents	0.511	0.655	0.185	2.314

Outcome: Venous events only (DVT and PE).

We examined the effects of HCQ treatment on mortality. Among the study patients, 159 died (about 31%) before 2013. The mortality rate in the HCQ-treated group was 22.4% compared to 38.5% among the non-treated group. However, a Cox regression with time dependent covariate of HCQ treatment showed no significant protective effect of HCQ treatment for mortality- HR = 0.851 95% CI 0.589 to 1.230.

Age (HR = 1.036 95% CI 1.020 to 1.053) and CRP levels (HR = 1.123 95% CI 1.077 to 1.171) were a significant risk factor for mortality while obesity (HR = 0.499 95% CI 0.305 to 0.817) showed a protective effect.

In addition we examined the effects of HCQ treatment dose on any cardiovastular disease. We constructed a regression model for patients treated with a dose of 400 mg/day vs non- treated patients and patients treated with 200 mg/day of HCQ vs non-treated patients.

We found that HCQ treatment with 400 mg/day dose showed a significant protective effect for any cardiovascular events (HR = 0.432 95% CI 0.243 to 0.768). The protective effect of the higher dose of 400 mg/day of HCQ was also significant for arterial events (HR = 0.433 95% CI 0.227 to 0.827) but not for venous thrombotic events or mortality.

However, the HCQ treatment with a dose of 200mg/day showed no significant protective effect for any cardiovascular disease (HR = 0.665 95% CI 0.349 to 1.269). This dose of HCQ also showed no significant protective events for arterial events, venous thrombotic events and mortality.

## DISCUSSION

These findings suggest that HCQ for treating rheumatoid arthritis significantly reduces the risk for cardiovascular morbidity. Patients who received 400 mg a day of HCQ had a lower incidence any cardiovascular events as well as arterial events(MI and stroke), while a dose of 200 mg a day of HCQ showed no significant protective effect. Furthermore, HCQ treatment had some effect on mortality rates, but it was not significant after the regression model was introduced. There was no significant effect of HCQ treatment duration on mortality. These effects were independent of other RA treatments evaluated.

This study shows a potential protective effect of HCQ on cardiovascular morbidity among RA patients. Cardiovascular disease is a grave concern for RA patients who are 1.5 times more likely to suffer from MI or stroke than the general population [19, 20]. The current strategy used to combat this risk includes adequate control of disease activity and addressing cardiovascular risk factors. Our findings suggest the possibility of repurposing the use of HCQ to reduce cardiovascular morbidity among RA patients. This could add additional clinical considerations for the physician in the choice of treatment for patients with RA. This is a preliminary study and additional research is needed to evaluate this effect.

The protective effect of HCQ demonstrated in this analysis is in line with two previous studies among lupus patients [21, 22]. It is also backed by evidence that HCQ has a positive effect on lipid profiles and diabetes [14–17]. We would like to note that a study published in 2006 by van Halm examined the effect of different combinations of DMARDs and did not show a protective effect for HCQ alone. This might be due to the different study design that examined the use of HCQ separately for only 36 patients [13].

Additional findings not included in the study design are a significant protective effect of MTX on cardiovascular disease. This result is similar to previous research [23].

Some limitations to this study must be taken into account. First, this is retrospective cohort study and several parameters of disease activity were not available for most of the patients, especially the DAS28 score. As a result, some of the differences between the groups could be attributed to variations in disease activity not detected by this analysis. Despite the importance of the DAS28 score in clinical assessment, two prospective studies failed to demonstrate a significant correlation between the score and CVD risk [24, 25]. Second, fewer patients received the lower daily dose of 200 mg HCQ than the 400 mg dose, potentially leading to less significant results among these patients. Third, this study was conducted among patients treated at one medical center, which might have caused an unknown selection bias.

The mechanism by which HCQ influences the risk for CVD events has been poorly investigated. Two small studies showed an improvement in arthrosclerosis among SLE patients [26, 27]. while other studies did not show this effect [28, 29]. In addition, a recent study suggests that HCQ possesses certain anti-platelet properties through the arachidonic acid pathway [30].

In conclusion, RA patients are at significant risk for cardiovascular disease and better measures are needed to cope with this hazard. This study suggests that HCQ, a commonly used and relatively safe drug, has a significant protective effect against cardiovascular mortality. This finding provides an additional clinical consideration for the attending physician in the choice of appropriate treatment for patients with RA.

## MATERIALS AND METHODS

### Study design

This was a retrospective cohort study among RA patients treated at Meir Medical Center in Kfar Saba, Israel from 2003 through 2013. All data were obtained manually by reviewing patient histories, discharge summaries and medical records using the hospital electronic archive. The Institutional Ethics Committee approved the study.

Inclusion criteria were a diagnosis of rheumatoid arthritis, age 18 years or older and sufficient follow up information. A total of 514 RA patients met the inclusion criteria. The patients were divided into two groups. The treated group consisted of 241 patients who were treated with HCQ at some point in the course of their disease. The non-treated group consisted of 273 patients who had never received the drug.

### Confounding factors

Data were collected regarding factors that could confound the effect of HCQ treatment on cardiovascular morbidity. The confounding factors were categorized as 1) demographic characteristics, 2) disease severity indicators, 3) common cardiovascular risk factors and protective treatment and 4) other treatment prescribed for RA.

The use of biological agents for the treatment of RA has been growing in Israel. However, many patients are still treated with traditional DMARDs alone. The small treatment rates precluded separate analysis of each biological drug and they were grouped together for statistical analysis.

### End points

Patient files were examined for incidence of cardiovascular events. For the non-treated group, only events that occurred after the diagnosis of RA were recorded. For the treated group, the events recorded occurred after initiation of HCQ treatment.

Data regarding arterial events including myocardial infarction (MI), and cerebrovascular events including stroke and transient ischemic attacks (TIA) were collected, as well as venous thrombosis events, including deep venous thrombosis (DVT) and pulmonary embolism (PE). A few ischemic colitis events and peripheral artery disease were recorded. The secondary study endpoint was any cause mortality.

### Statistical analysis

To evaluate the difference between patients treated with HCQ and those who did not, we applied the T test for continuous variables and chi-square test for categorical variables.

Multivariate models were adjusted for age and other known risk factor for cardiovascular disease. We used the time-dependent Cox proportional hazards regression model to estimate the risk for CV event among rheumatoid arthritis patients according to HCQ use. Hazard ratios (HRs) and 95% confidence intervals (CIs) were evaluated to determine the association between the risk of CV event and HCQ use.

As not all the patients started to use HCQ from the beginning of the follow-up period, the drug use varied over time, it was measured as a time-dependent covariate in the Cox proportional hazard model. We used time-dependent variables for present age and present disease years as well.

We performed three different comparisons: treated versus non-treated patients, patients treated with 200 mg/day versus non-treated patients, and patients treated with 400 mg/day versus non-treated patients.

The SPSS software (Version 24.0, IBM SPSS Statistics) was used for data management and statistical analysis. A 2-sided P value < .05 was considered statistically significant.
